# Is the Prevalence of Equinus Foot in Cerebral Palsy Overestimated? Results from a Meta-Analysis of 4814 Feet

**DOI:** 10.3390/jcm10184128

**Published:** 2021-09-13

**Authors:** Axel Horsch, Matthias C. M. Klotz, Hadrian Platzer, Svenja Seide, Nancy Zeaiter, Maher Ghandour

**Affiliations:** 1 Department of Orthopedics and Trauma Surgery, Heidelberg University Hospital, 69118 Heidelberg, Germany; hadrian.platzer@med.uni-heidelberg.de (H.P.); dr.ghandour@hotmail.com (M.G.); 2 Marienkrankenhaus Soest, Orthopedics and Trauma Surgery, 59494 Soest, Germany; mcmklotz@gmx.net; 3 Institute of Medical Biometry, University of Heidelberg, 69120 Heidelberg, Germany; seide@imbi.uni-heidelberg.de; 4 Department of Plastic Surgery, Al Zahraa Hospital University Medical Center, Beirut 1003, Lebanon; zeaiternancy@gmail.com

**Keywords:** cerebral palsy, equinus foot, prevalence, meta-analysis

## Abstract

Background: Equinus is a common foot deformity in patients with cerebral palsy (CP). However, its prevalence is scarcely reported in the literature. Therefore, we conducted this review to estimate the prevalence of equinus foot in CP. Methods: Eight databases were searched. Our primary outcome was the prevalence of equinus foot in CP patients. Subgroup analysis was conducted based on study design, the laterality of CP, and whether equinus foot was defined or not. Results: The prevalence of equinus foot in CP was 93% (95% CI: 71–99). The prevalence was 99% (95% CI: 55–100), 96% (95% CI: 57–100), and 65% (95% CI: 37–86) in unilateral, both, and bilateral CP, respectively. Based on study design, equinus foot prevalence was 92% (95% CI: 34–100) in case series and 62% (95% CI: 47–74) in cohort studies. Four studies reported definition criteria for equinus foot, with a pooled prevalence rate of equinus foot of 99% (95% CI: 36–100) compared to a rate of 89% (95% CI: 59–98) among studies that lacked a definition criterion. Conclusions: This is the first meta-analysis to address the prevalence of equinus foot in CP patients. Although its prevalence is very high, our findings should be interpreted with caution due to the presence of multiple limitations, such as the lack of standardized definition criteria for equinus foot, the inappropriate study design, the wide confidence interval of equinus foot rate, and the small number of studies investigating it as a primary outcome.

## 1. Introduction

Cerebral palsy (CP) is known as a heterogenous group of neuromotor disorders that affect the brains of newborn infants or developing fetuses. These disorders occur at an early age in a non-progressive manner [1]. CP is perceived as one of the most frequent causes of physical disability among children [1,2]. Its prevalence rate has been consistent throughout the years. A recent meta-analysis of 49 studies reported an overall estimated prevalence rate of CP of 2.11 per 1000 live births (with a 95% confidence interval of 1.98–2.25) [3].

Equinus is the most common foot deformity in patients with cerebral palsy (CP). It is quickly recognized as soon as the children start standing or walking. It appears as failure to perform sufficient ankle dorsiflexion to allow heel contact with the supporting surface without the need for compensatory lower and foot biomechanics. Underlying factors that lead to equinus foot are weak muscle power, muscle imbalance, spasticity, and muscle and/or ankle joint contracture of adjunct joints. While it is initially mostly a dynamic contracture (due to muscle imbalance), it may develop into a fixed contracture attributed to soft-tissue, articular, and bone changes. These changes lead to functional impairment resulting in an unbalanced gait and increased risk of trips and falls. It can also reduce walking and exercise tolerance and even cause long-term bone and soft-tissue deformity. When gait, posture, or stability is compromised, intervention is indicated. That being said, there are no available resources that accurately report the prevalence of equinus foot among patients with CP.

Also, in the literature, a wide variety of definitions for equinus foot exist, and this contributes to the difficulty in clearly understanding this disease entity. In some studies, equinus foot is defined as inability of foot/ankle dorsiflexion above plantigrade, with the hindfoot in a neutral position, while the knee is extended [4,5]. In other studies, equinus foot is defined as ≤5° of ankle dorsiflexion while the knee is extended [6] or when the degree of ankle dorsiflexion during the stance phase of gait is >1 standard deviation (SD) less than the average reference value [7]. Moreover, in a previous consensus workshop, equinus foot was defined as the presence of a functional gait or standing pattern that is characterized by unequal weight bearing on the metatarsal heads, along with increased plantarflexion at the ankle [8]. However, based on the aforementioned definition, the findings of many research studies in this particular field cannot be interpreted equally or be generalizable to the overall population.

Therefore, we conducted this systematic review and meta-analysis to determine the overall prevalence of equinus foot deformity among patients with CP. We also aimed to investigate whether or not there is a standardized definition criterion for equinus foot in the literature, and whether or not it would affect the overall estimation of the prevalence of equinus foot in CP patients.

## 2. Materials and Methods

### 2.1. Search Strategy and Study Selection

The study process was conducted following the accepted methodology recommendations of the PRISMA checklist for systematic review and meta-analysis where registration of the protocol is not mandated [9]. A systematic electronic database search was conducted for relevant studies published from inception and till 29 June 2021 in eight databases: PubMed(NCBI, Bethesda, MD, USA), Scopus (ELSEVIER, Amsterdam, Netherlands), Science Direct (ELSEVIER, Amsterdam, Netherlands), the New York Academy of Medicine (NYAM, New York, NY, USA), Virtual Health Library (VHL, BIERME/PAHO/WHO, Sao Paulo, Brazil), the WHO Global Health Library (GHL, BIERME/PAHO/WHO, Sao Paulo, Brazil), clinicaltrials.gov (NLM, Bethesda, MD, USA), and the Cochrane Controlled Register of Trials (CENTRAL, John Wiley & Sons Ltd., Hoboken, NJ, USA).

The search was conducted using keywords (equinus) AND (cerebral palsy OR CP OR gait pattern) AND (prevalence OR rate OR incidence) and/or medical subject (MeSH) terms, as appropriate. We further did a manual search of references in our included papers to avoid missing relevant studies [10,11]. The manual search was conducted by going through the references of relevant articles and by using the “similar articles” option in PubMed, relating to the studies that were finally included in our review.

The search process was done based on the PICO framework: participants were any patients with cerebral palsy that was diagnosed at any time-point (children or adults), no particular intervention was studied, no comparison group was included, and the prevalence of equinus foot deformity was our outcome of interest. We included studies regardless of their study design. On the other hand, research papers were excluded if they were consistent with one of the following exclusion criteria: non-original studies; non-human (in vitro or animal) studies; duplicate records; overlapped data; cases where data could not be reliably extracted, or incomplete reports; abstract-only articles; reviews; theses; books; and conference papers or articles without available full texts.

The title and abstract screenings were performed by four independent reviewers. Then, three independent reviewers performed a full-text screening to ensure the inclusion of relevant papers in our systematic review. Any disagreement was resolved by discussion and referring to the senior author when necessary.

### 2.2. Data Extraction

Two authors developed the data extraction sheet using Microsoft Excel software (Microsoft, Albuquerque, NM, USA). Data extraction was performed by three independent reviewers using the Excel sheet. Three reviewers extracted the data and did the quality assessment of all articles. The fourth independent reviewer performed data checking to ensure the accuracy of the extracted data. All disagreements and discrepancies were resolved by discussion and consultation with the senior author when necessary. The extraction sheet was made up of three major categories: baseline information of included studies (i.e., article ID, title, journal name, country, last name of first author, study design, number of included patients), characteristics of included studies (i.e., age, gender, laterality of CP, CP type, equinus foot type and definition, intervention type, and follow-up period), and outcomes data (prevalence of equinus foot). The definition of equinus foot was considered a secondary outcome, in order to determine whether or not there is a standardized definition criterion for equinus foot.

### 2.3. Risk of Bias

Three independent reviewers evaluated the risk of bias in included studies. We used the National Institute of Health (NIH) quality assessment tool for observational cohort and cross-sectional studies [12], case series, case-control studies, and clinical trials. Any discrepancy between the reviewers was solved by discussion.

### 2.4. Statistical Analysis

Overall pooled proportions were estimated using a generalized linear mixed and a logit transformation to pool the prevalence [13]. Prevalence was pooled using a random-effect meta-analysis with the odds ratio as effect measure and the DerSimonian–Laird estimator for between-trial heterogeneity [14]. All results were visualized using forest plots. Subgroup analyses are performed for laterality, study design, and the definition of equinus foot for both endpoints, and in addition for different intervention types for the prevalence of equinus foot. Between-trial heterogeneity was reported by using the estimated Q and the $I^{2}$ test statistics. Analyses were performed using R [15] version 4.0.3 and its extension meta (R Foundation for Statistical Computing, Vienna, Austria) [16].

## 3. Results

### 3.1. Search Results

**Figure 1:**
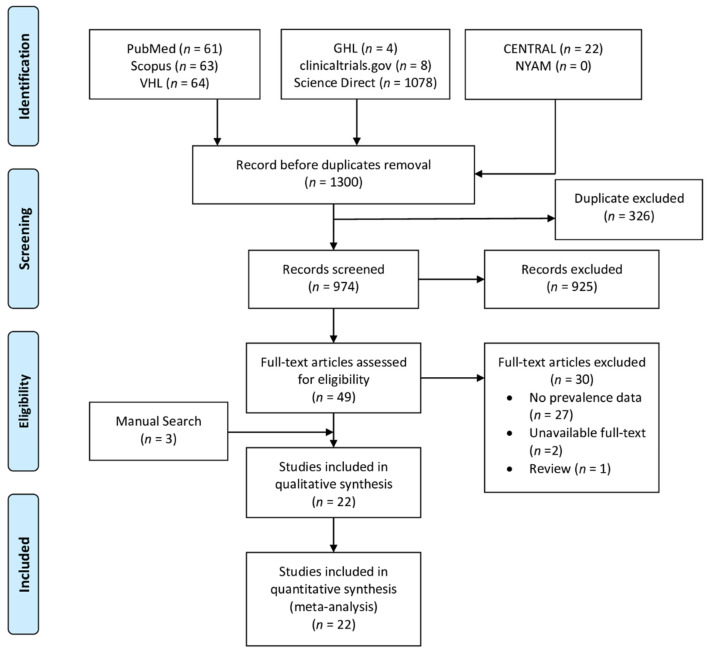
PRISMA diagram showing the screening process of our review.

The database search yielded 1300 studies based on our search criteria. A total of 974 studies were included in the title and abstract screening phase after the removal of duplicates. Forty-nine studies were finally included in the full-text screening phase, out of which 30 studies were excluded. Upon doing the manual search of relevant articles, we found three articles that were consistent with our eligibility criteria. Finally, a total of 22 studies were included in the systematic review and meta-analysis. The process of study screening and selection is reported in the PRISMA diagram (Figure 1).

### 3.2. Study Characteristics

The baseline characteristics of included studies are reported in detail in Table 1. A total of 22 studies were finally included in the analysis. Based on the study design, 13 were retrospective cohort, 4 were case series, 1 was case control, 1 was cross-sectional, 1 was prospective cohort, 1 was randomized controlled trial, and 1 was a single-armed prospective clinical trial.

The number of patients with cerebral palsy varied substantially among the included studies, ranging from 7 patients to 1147 patients. Seventeen studies reported the type of CP among the included patients: 2 studies included patients with mixed CP, and 15 studies included patients with spastic CP.

According to the type of equinus foot, 3 studies reported patients with dynamic equinus, one study reported patients with fixed equinus, and 18 studies did not report the type of equinus foot. The mean age of patients with CP ranged from 3.8 (SD = 2) years [17] to 18.2 (SD = 9.9) years [6]. Based on gender, male patients with CP ranged from 33% [24] to 75% of the studied population [29].

## 4. Prevalence of Equinus Foot

**Figure 2:**
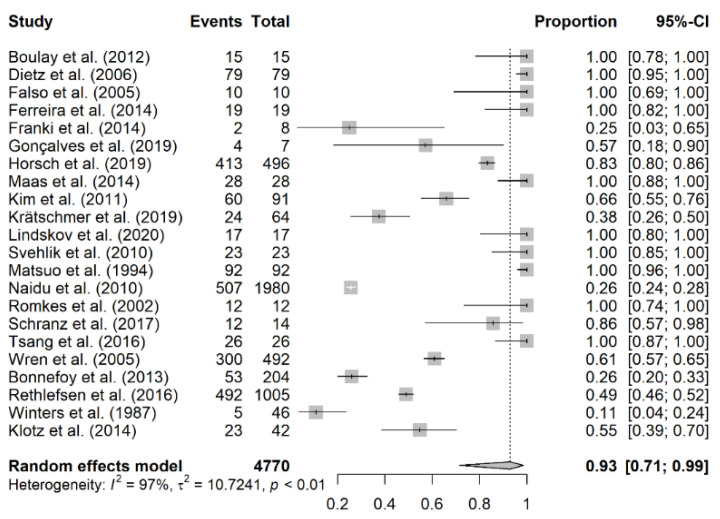
The pooled prevalence of equinus foot in cerebral palsy.

The prevalence of equinus foot was calculated based on the number of equinus foot per affected limb and not by the affected individual (Table 2). The overall number of included patients was 3595, with 4814 affected limbs being analyzed. The prevalence of equinus foot among patients with cerebral palsy varied substantially among included individual studies, ranging from 10.9% [35] to 100% [7,17,18,19,20,23,26,27,29,31]. A total of 22 studies were included in the meta-analysis with an overall prevalence rate of equinus foot of 93% (95% CI: 71–99%; *I*^2^ = 100%, *p* < 0.01) (Figure 2). We further did a subgroup analysis of the prevalence of equinus foot in patients with CP based on laterality of CP, study design, and the definition of equinus foot (defined/not defined).

### 4.1. Prevalence of Equinus Foot Based on Laterality

**Figure 3:**
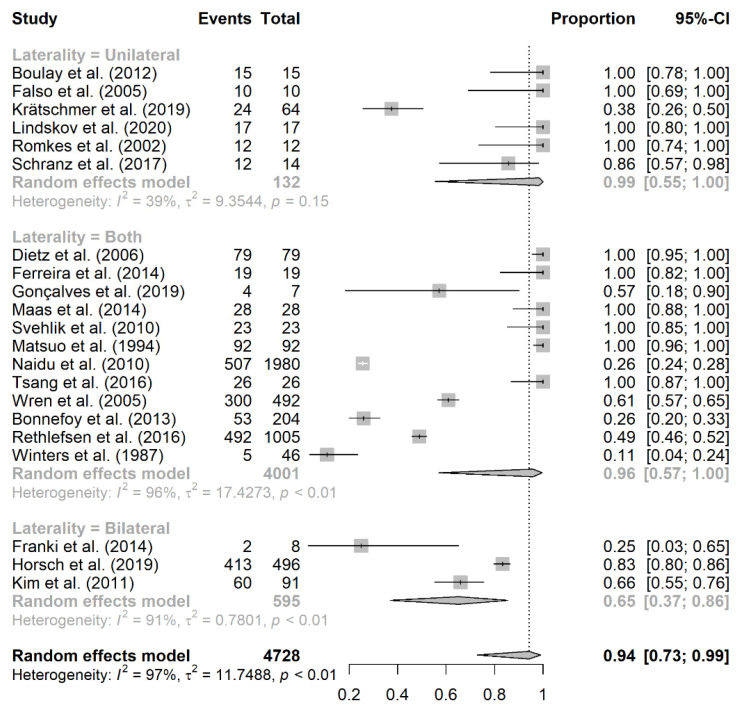
Subgroup analysis of equinus foot prevalence based on the laterality of cerebral palsy.

Based on laterality, 6 studies reported unilateral CP, with an overall prevalence rate of 99% (95% CI: 55–100%; *I*^2^ = 92%, *p* = 0.15). A total of 12 studies reported patients with both unilateral and bilateral CP, with an overall prevalence rate of equinus foot of 96% (95% CI: 57–100%; *I*^2^ = 100%, *p* < 0.01). On the other hand, only 3 studies reported patients with bilateral CP, with an overall prevalence rate of 65% (95% CI: 37–86%; *I*^2^ = 93%, *p* < 0.01) (Figure 3).

### 4.2. Prevalence of Equinus Foot Based on Study Design

**Figure 4:**
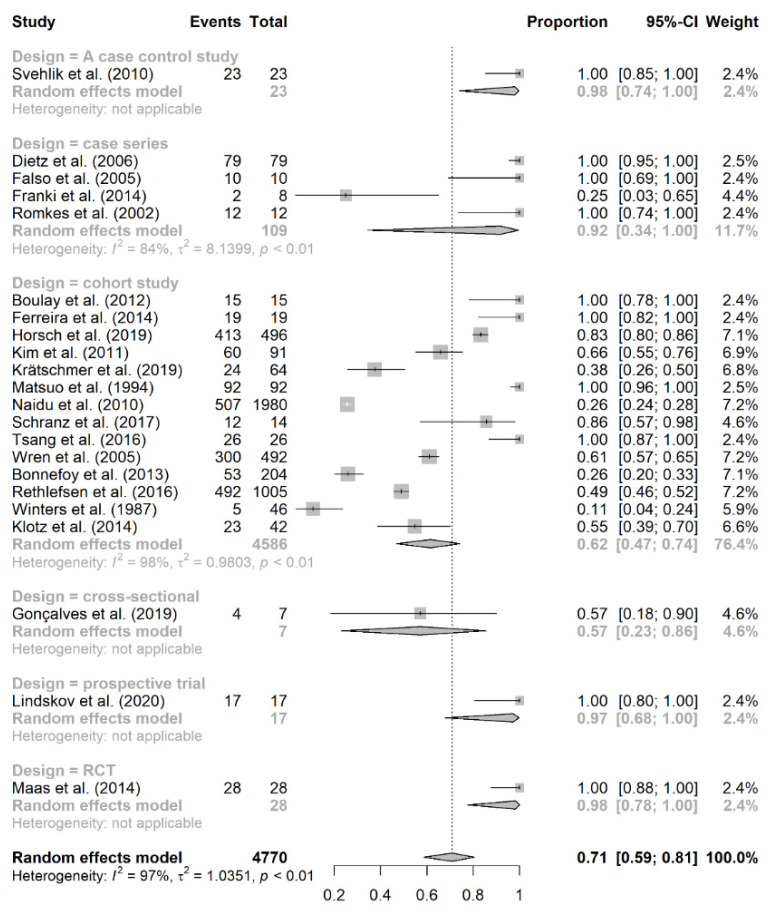
Subgroup analysis of equinus foot prevalence based on study design.

The subgroup analysis of prevalence of equinus foot based on study design is presented in Figure 4. Certain designs had only one study, and therefore, they were not reliable in estimating the overall prevalence rate of equinus foot in CP. Meanwhile, 14 studies were cohort in design, and they had an overall prevalence rate of 62% (95% CI: 47–74%; *I*^2^ = 98%, *p* < 0.01) [6,17,20,24,25,27,28,30,31,32,33,34,35,36]. On the other hand, 4 studies were case series in design, with an overall prevalence rate of 92% (95% CI: 34–100%; *I*^2^ = 84%, *p* < 0.01) [18,19,21,29].

### 4.3. Prevalence of Equinus Foot Based on the Definition of Equinus

**Figure 5:**
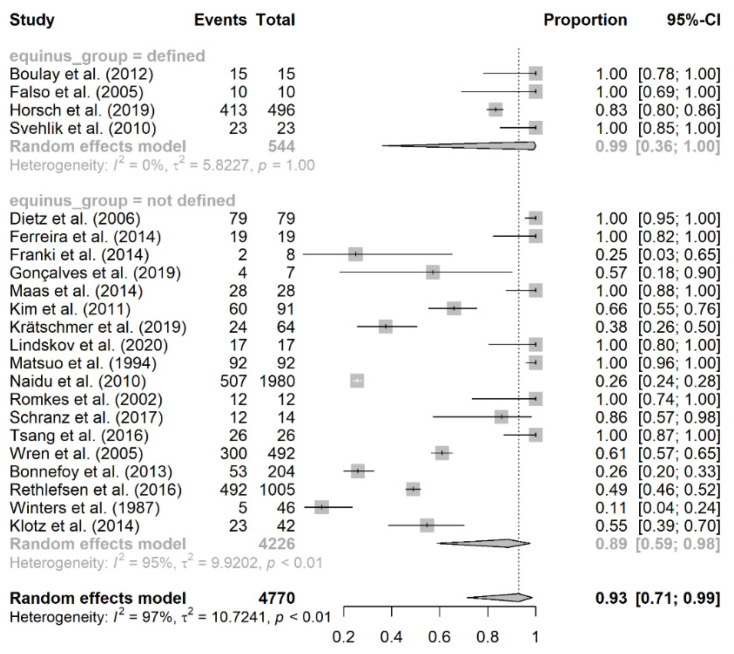
Subgroup analysis of equinus foot prevalence based on the presence/lack of definition criteria for equinus.

A total of 4 studies reported a definition criterion for equinus foot, and these studies had an overall prevalence rate of 99% (95% CI: 36–100%; *I*^2^ = 85%, *p* = 1.00) [6,7,17,19]. On the other hand, 18 studies did not report a definition criterion for equinus foot, and they had an overall prevalence rate of 89% (95% CI: 59–98%; *I*^2^ = 93%, *p* < 0.01) (Figure 5).

### 4.4. Risk of Bias

Quality assessment of the included studies was conducted by the NIH quality assessment tool for each distinctive study design. A total of 16 studies were of fair quality (moderate risk of bias) [6,7,17,20,22,24,25,28,30,31,32,33,34,35,36,37], 4 studies were of poor quality (high risk of bias) [18,19,21,29], and 2 studies were of good quality (low risk of bias) [23,26].

## 5. Discussion

To the best of our knowledge, this is the first systematic review and meta-analysis to estimate the pooled prevalence of equinus foot deformity among patients with CP. A total of 22 studies were finally included in our analysis. Among the included studies, the majority were cohort in design (13 retrospective and 1 prospective cohort studies), followed by case series (4 studies), case control (1 study), single-armed clinical trial (1 study), cross-sectional (1 study), and randomized controlled trial (1 study). The overall number of included patients with CP in our study is 3595 patients and 4814 limbs/feet, ranging from 7 patients in the study of Goncalves et al. [22] to 1147 patients in the study of Naidu et al. [28]. Most of the included studies reported the data of patients with the spastic type of CP (15 studies) [6,7,19,20,21,22,23,24,25,26,27,28,30,31,35]. The age of CP patients among included studies ranged from a mean of 3.8 years (with SD of 2) in the study of Boulay et al. [17] to 18.2 years (with SD of 9.9) in the study of Horsch et al. [6]. Males constituted the majority of the studied population, with an overall rate of 59.78%, while ranging from 33% in the study of Kim et al. [24] to 75% in the study of Romkes et al. [29].

In our study, the pooled prevalence of equinus foot in patients with CP was 93% (95% CI of 71-99). The analysis yielded a significant substantial degree of heterogeneity. The prevalence of equinus foot in CP varied significantly among included individual studies, ranging from 10.9% in the study of Winters et al. [35] to 100% in other studies [7,17,18,19,20,23,26,27,29,31].

We also aimed to determine whether the rate of equinus foot in CP would differ based on the following factors: laterality of CP, study design, and whether or not equinus foot was defined. The reason why we did a subgroup analysis based on study design is because cross-sectional studies are known as the optimum option for determining prevalence; however, only one of the included studies was cross-sectional in design [22]. Therefore, we conducted this analysis to determine whether the prevalence would change based on the change in study design or not. Upon estimating the prevalence of equinus foot based on the laterality of CP, we found that patients with bilateral CP had lower prevalence of equinus foot compared to unilateral and combined CP (65% vs. 99% vs. 96%), respectively. Notably, the analysis revealed a significant substantial degree of heterogeneity. This could be related to the differences among the included studies at baseline. For instance, there were only 3 studies that reported bilateral CP, and the study of Franki et al. [21] had a prevalence rate of 25% (2 equinus foot cases out of 8 CP patients), which could be the result of the underestimation of equinus foot prevalence among CP patients. Also, the study design of these studies could account for the heterogeneity observed among the included studies.

The subgroup analysis based on study design revealed that cohort studies had much lower prevalence rate compared to case series (62% vs. 92%). Other study designs were not appropriate for subgroup analysis due to the lack of enough studies to carry out this analysis. It should be noted that the results of the cohort subgroup could be more reflective of the actual prevalence rate of equinus foot in CP because the design of such studies is more appropriate than that of case-series studies, the limited case numbers of which could cause over- or underestimation of the true prevalence rate. Of note, the confidence interval of equinus foot prevalence in each subgroup is wide. For example, the 95% CI of equinus foot in cohort studies ranged from 47 to 74%, indicating that the true value could lie anywhere between these values. Therefore, we are not truly confident that the reported pooled prevalence accurately reflects the true population value.

There is a clear lack of a standardized definition criteria for equinus foot in the literature. In our study, we found that only 4 studies reported a definition criterion for equinus foot [6,7,17,19], while the remaining studies did not define equinus foot in their populations. Based on whether or not the studies reported a definition criterion for equinus foot, we carried out a subgroup analysis, which revealed a slight difference in the prevalence rate of equinus foot based on the presence of definition criteria or not (89% vs. 99%), respectively. Of note, the criteria that was used to define equinus foot was different. It was defined as an ankle planter flexion below 0° in the study of Boulay et al. [17], while in the study of Horsch et al. [6], it was defined as ≤5° of ankle dorsiflexion during knee extension. Meanwhile, in the study of Svehlik et al. [7], equinus foot was defined when the peak ankle dorsiflexion during the stance phase of gait was >1SD less than the average reference value. The authors of this study even provided a clear definition criterion based on the type of equinus foot, where fixed equinus was defined as a maximum dorsiflexion of the ankle <5° when the knees were fully extended under general anesthesia. In the study of Falso et al. [19], equinus foot was defined as ankle plantarflexion of 1SD below the mean of normal range during the stance phase, regardless of the presence or absence of an accompanying hindfoot and/or forefoot varus or valgus.

Equinus foot is distinguished by decreased ankle joint dorsiflexion, although there is a lack of consensus on the exact definition and diagnostic criteria. The static ankle joint equinus foot represents a reduced range of dorsiflexion at the ankle joint. However, there is no agreement as to what degree of dorsiflexion reduction is needed for this condition to constitute. This led physicians to use a wide variety of restrictions for diagnosis of dorsiflexion [38]. Sobel et al. [39] suggested that patients with less than 0° of dorsiflexion can be diagnosed with equinus foot (i.e., no step beyond plantigrade), while Orendurff et al. [40] recommended a cut-off value of 5°. Again, DiGiovanni et al. [41] proposed a minimum value of fewer than 10 degrees of dorsiflexion. This is consistent with the need for at least 10 degrees of dorsiflexion to maintain normal gait and prevent possible increased forefoot loading throughout locomotion [40,42]. This proposal is consistent with Meyer’s more recent recommendation that, instead of basing an equinus foot diagnosis on a specific range of dorsiflexion motion, a diagnosis should be verified when there is a reduction in dorsiflexion of a magnitude. Stress increases on the Achilles tendon and loads simultaneously on the forefoot [43]. There is no evidence to support that developing foot or lower-leg equinus foot is attributed to raising forefoot pressure during locomotion to base an equinus foot diagnosis on a limit of 10° of dorsiflexion. Moreover, although a cut-off point of 10° may increase forefoot loading during locomotion, Orendurff et al. [40] proposed that a dorsiflexion range of 5° be used for the diagnosis of equinus foot as they found that forefoot pressure was higher in patients with less than 5° dorsiflexion compared to patients with more than 5° dorsiflexion (*p* < 0.05).

Accordingly, guidance is presented for restricting dorsiflexion to either 5° or 10° as the basis for an equinus foot diagnosis. This marks a 10° cut-off point based on gait adjustments that may increase forefoot loading and a 5° cut-off point based on a higher degree of forefoot load. As greater limitations in the range of motion for dorsiflexion increase forefoot loading, there exists a lack of understanding is present regarding the threshold reduction leading to an increase in forefoot pressure necessary to facilitate the development of foot or lower-leg abnormalities. Besides, diagnosis of equinus foot should be based on criteria anticipating the development of foot or lower-leg abnormalities. It is also difficult to establish a reasonable range of motion beyond which a description of equinus foot may be justified without any prospective research to evaluate the effects of various rates of dorsiflexion restriction on forefoot loading and the long-term effects of this loading on foot safety. These prospective studies are urgently required to investigate the relation between limitations in the range of motion for dorsiflexion and the subsequent development of defects in the foot or lower legs. In the absence of conclusive data, if we use a credible and accurate method, we suggest a two-stage description based on the evidence currently available. The first stage would represent dorsiflexion of less than 10°, indicating minor compensation and lower forefoot pressure. The second would represent dorsiflexion of less than 5°, indicating major compensation and greater forefoot pressure.

In addition to the two-stage approach, we propose that the following additional qualifications will be of great importance for a thorough definition and diagnosis of equinus foot: (1) physical examination (i.e., estimating the tone, the range of motion at the ankle, weakness, fixed contracture of the heel cord, etc.), (2) gait/motion analysis (either video-guided, instrumented, or others), (3) the site of the lesion, (4) the etiology, and (5) estimating the age of the patients at the time of the lesion occurrence and also at the time of clinical presentation.

## 6. Limitations

Although our study is the first to report the pooled prevalence rate of equinus foot among patients with CP, several limitations were encountered. First, and most importantly, equinus foot was not considered a primary outcome in the majority of included studies; it was reported as one of the gait patterns that are encountered in patients with CP. Also, the design of some included studies is not appropriate to estimate the actual prevalence rate of equinus foot in CP. Therefore, future studies should be conducted as cross-sectional or prospective cohort design to accurately examine the prevalence of gait abnormalities, especially equinus foot, in patients with cerebral palsy following their diagnosis. Second, there is a clear lack of a standardized definition criterion for equinus foot, which could bias the reported rate in our study. Third, the study designs of the included studies were different and this could reflect and explain the reason for encountering the substantial heterogeneity in the primary and subgroup analyses. Finally, the confidence interval of the reported prevalence of equinus foot is very wide, which reflects the inaccuracy of such estimates. Therefore, our findings should be interpreted with caution and should not be generalizable to the overall population. As a result, more studies of appropriate study design, sample size, and definition criteria are still warranted in order to accurately estimate the true rate of equinus foot in CP patients.

## 7. Conclusions

The pooled prevalence rate of equinus foot among patients with cerebral palsy is 93%. However, this rate could be an overestimation of the true prevalence of equinus foot due to a number of reasons: the lack of standardized definition criteria for equinus foot, the inappropriate study design, the wide confidence interval of equinus foot rate, and the small number of studies investigating this matter as a primary outcome. Therefore, more studies are still warranted to reach a more definitive conclusion in this matter.

## Figures and Tables

**Table 1:** Baseline characteristics of studies reporting equinus foot among patients with cerebral palsy (*n* = 22).

Author/YOP	Laterality	Study Design	Sample	CP Type	Equinus Type	Intervention Type	Age Mean (SD)	Male (%)	Follow-Up (Months)
Boulay, 2012 [17]	unilateral	Prospective cohort	15	NA	dynamic	NA	3.8 (2)	60%	NA
Dietz, 2006 [18]	Both	Retrospective case series	79	Mixed	NA	TAL	7.25 ^$^	NA	84
Falso, 2005 [19]	Unilateral	Case series	10	spastic	dynamic	BTX-A	9 ^$^	70%	1
Ferreira, 2014 [20]	Both	Retrospective cohort	19	spastic	NA	Gastrocnemius fascia lengthening	8 ^$^	68.40%	13
Franki, 2014 [21]	Bilateral	Case series	8	spastic	NA	NA	NA	NA	NA
Gonçalves, 2019 [22]	Both	Cross-sectional	7	spastic	NA	NA	8.6 ^$^	57.10%	NA
Horsch, 2019 [6]	Bilateral	Retrospective cohort	248	spastic	fixed	NA	18.2 (9.9)	62.90%	NA
Maas, 2014 [23]	Both	RCT	28	spastic	NA	Knee–ankle–foot orthosis	8.6 (3.2)	53.60%	12
Kim, 2011 [24]	Bilateral	Retrospective cohort	91	spastic	NA	NA	10.4 ^$^	33.00%	NA
Krätschmer, 2019 [25]	Unilateral	Retrospective cohort	64	spastic	NA	NA	4–17 ^#^	NA	NA
Lindskov, 2020 [26]	Unilateral	Prospective trial	17	spastic	NA	Ankle–foot orthosis	8.4 ^$^	64.70%	NA
Svehlik, 2010 [7]	Both	Case-control study	23	spastic	dynamic (63.3%) and fixed (38.7%)	NA	NA	NA	NA
Matsuo, 1994 [27]	Both	Retrospective cohort	92	spastic/athetoid	NA	Surgery	3–19 ^#^	NA	50.4
Naidu, 2010 [28]	Both	Retrospective cohort	1147	spastic	NA	BTX-A	4.6	NA	NA
Romkes, 2002 [29]	Unilateral	Case series	12	NA	NA	Ankle-foot orthosis	11.9	75%	NA
Schranz, 2017 [30]	Unilateral	Retrospective cohort	14	spastic	NA	Single-event multilevel surgery	12.1	64.30%	60
Tsang, 2016 [31]	Both	Retrospective cohort	26	spastic	NA	Multilevel surgery	16.8	53.80%	17
Wren, 2005 [32]	Both	Retrospective cohort	492	Mixed	NA	NA	9.6 (4)	54.90%	NA
Bonnefoy, 2013 [33]	Both	Retrospective cohort	122	NA	NA	NA	14.2 (7.5)	58.20%	NA
Rethlefsen, 2016 [34]	Both	Retrospective cohort	1005	NA	NA	NA	9 (5) *	58.60%	NA
Winters, 1987 [35]	Both	Retrospective cohort	46	Spastic	NA	NA	15 (12)	NA	NA
Klotz, 2014 [36]	Both	Retrospective cohort	37	NA	NA	NA	NA	62.20%	NA

Originally, data for age were reported in the form of means and their corresponding standard deviation (SD) *: Age is reported as median and interquartile range (IQR); ^#^: Age is reported as range alone; ^$^: Age is reported as a mean without SD; CP: Cerebral palsy; YOP: year of publication; NA: not available; BTX-A: Botulinum Toxin-A; TAL: tendo-Achilles lengthening.

**Table 2:** Prevalence of equinus foot per patient and per foot among the included studies of patients with cerebral palsy.

Author/YOP	Prevalence per Patient		Prevalence per Limb	
	N	T	N	T
Winters, 1987 [35]	5	46	5	46
Franki, 2014 [21]	2	8	2	8
Naidu, 2010 [28]	NR	1147	507	1980
Bonnefoy, 2013 [33]	NR	122	53	204
Krätschmer, 2019 [25]	24	64	24	64
Rethlefsen, 2016 [34]	492	1005	492	1005
Klotz, 2014 [36]	NR	30	23	42
Gonçalves, 2019 [22]	4	7	4	7
Wren, 2005 [32]	300	492	300	492
Kim, 2011 [24]	60	91	60	91
Horsch, 2019 [6]	NR	248	413	496
Schranz, 2017 [30]	12	14	12	14
Boulay, 2012 [17]	15	15	15	15
Dietz, 2006 [18]	79	79	79	79
Falso, 2005 [19]	10	10	15	15
Ferreira, 2014 [20]	19	19	29	29
Maas, 2014 [23]	28	28	36	36
Lindskov, 2020 [26]	17	17	17	17
Svehlik, 2010 [7]	23	23	31	31
Matsuo, 1994 [27]	92	92	92	92
Romkes, 2002 [29]	12	12	12	12
Tsang, 2016 [31]	26	26	39	39

YOP: year of publication; NR: not reported: N: number of equinus cases; T: total number of cases/limbs in the sample.

## Data Availability

All of the data analyzed in this manuscript can be provided upon request by contacting the corresponding author.
